# Health Tract, No. 3

**Published:** 1865

**Authors:** 


### HEALTH TRACT, No. 3.

### HOW TO CURE A COLD.

The moment a man is satisfied he has taken cold, let him do three things:

First, eat nothing; second, go to bed, cover up warm in a warm room; third, drink as much cold water as he can, or as he wants, or as much hot herb-tea as he can; and in three cases out of four he will be almost well in thirty-six hours.

If he does nothing for his cold for forty-eight hours after the cough commences, there is nothing that he can swallow that will, by any possibility, arrest the cold, for, with such a start, it will run its course of about a fortnight in spite of all that can be done, and what is swallowed in the mean time in the way of food, is a hindrance and not good.

"Feed a cold and starve a fever" is a mischievous fallacy. A cold always brings a fever; the cold never beginning to get well until the fever subsides; but every mouthful swallowed is that much to feed the fever; and but for the fact that as soon as a cold is fairly started, nature, in a kind of desperation, steps in and takes away the appetite, the commonest cold would be followed by very serious results, and in frail people would be always fatal.

These things being so, the very fact of waiting forty-eight hours gives time for the cold to fix itself in the system; for a cold does not usually cause cough until a day or two has passed, and then waiting two days longer gives it the fullest chance to do its work before any thing at all is done.

Intelligent druggists know that all medicines sold for coughs, colds, consumption, and tickling in the throat, contain opium in some form or other. They repress the cough but do not eradicate it; hence the first purchase paves the way for a second or a third; meanwhile, as it is the essential nature of opium to close up, to constringe, to deaden the sensibilities, the bowels do not feel the presence of their contents calling for a discharge, and constipation is induced and becomes the immediate cause of three fourths of all ordinary ailments, such as headache, neuralgia, dyspepsia, and piles.

Warmth and abstinence are safe and certain cures when applied early. Warmth keeps the pores of the skin open, and relieves it of the surplus which oppresses it; while abstinence cuts off the supply of material for phlegm, which would otherwise have to be coughed up.

